# Hidden Costs of Workflow Challenges in Endoscopy Units: Insights from a Multinational Survey

**DOI:** 10.1055/a-2879-7529

**Published:** 2026-06-10

**Authors:** Ulrike Beilenhoff, Christoph Schlag, Dörte Wichmann

**Affiliations:** 1 Scientific SecretaryEuropean Society of Gastroenterology and Endoscopy Nurses and Associates (ESGENA)UlmBWGermany; 2 Department of Gastroenterology and Hepatology 27243 UniversitätsSpital ZürichZürichZHSwitzerland; 3 Working Group of Expertimental Endoscopy Training and Development, University of TübingenTübingenBWGermany

**Keywords:** endoscopy nursing, reprocessing, single-use versus reusable

## Abstract

**Background** The daily work of endoscopy nurses involves a variety of tasks, both within and outside the endoscopy department. A better understanding of their workload can reveal opportunities for improving cost efficiency.

**Methods** A cross-sectional, international, 23-question anonymous online survey was administered in various languages via nursing associations to nurses in endoscopy departments in 33 countries.

**Results** Responses from 456 nurses specializing in gastrointestinal and pulmonary endoscopy were included in the analysis. The majority of respondents worked in large centers that perform a high volume of procedures and had many years of experience working in endoscopy. The tasks that nurses frequently performed included endoscope reprocessing (full cycle: 51%; on-call: 72%), and moving the endoscopy tower (daily: 23%; weekly: 43%; monthly: 20%; rarely/never: 14%). Over half (51%) of the nurses reported that patients were transported from other departments to the endoscopy unit on a weekly basis, with 21% reporting daily movement and 23% monthly. Delays in patient procedures due to a lack of available endoscopes were observed daily, weekly, or monthly by 62% of the respondents. The advantages of using disposable endoscopes cited by respondents included increased patient safety ( *n* = 306; 67%), availability ( *n* = 285; 63%), and increased staff safety ( *n* = 232; 51%).

**Conclusion** The evaluated survey revealed potential starting points for achieving efficiency improvements and cost savings. Of particular interest is reducing the time nursing staff spend on nonclinical tasks.

## Introduction

### Background

The range of tasks performed by endoscopy nurses has expanded over the past 30 years. As well as assisting with complex and sometimes invasive procedures, nurses perform a variety of nonclinical tasks. 1 Endoscopic procedures are also frequently performed outside the endoscopy department, for example in intensive care units (ICUs) and operating rooms (ORs). Nurses are often responsible for transporting equipment. Patient transport to and from the endoscopy unit may also be part of their responsibilities. In many hospitals, endoscope reprocessing is performed by endoscopy nurses. Nurses also perform administrative tasks such as coding, checking inventory, and ordering materials. These tasks require a significant amount of time from well-trained nurses.

Improving efficiency in endoscopy departments offers the opportunity to save costs and improve the quality of patient care, which benefits patients, staff, and payers. 2 To identify potential areas for improving operational and financial efficiency, it is necessary to capture the total workload of endoscopy nurses, including clinical and nonclinical tasks. Clinical nursing hours can be freed up for additional procedures, with costs being saved and additional revenue being generated as a result.

### Study Objective

This study aimed to improve understanding of the daily work routines of endoscopy nurses, with the intention of identifying potential cost and time savings and releasing clinical nursing hours. The study looked at the tasks of endoscopy nurses in their day-to-day work environment and evaluated the challenges of device handling, reprocessing, and stress factors.

## Patients/Materials and Methods

### Study Aims

Primary aim of the survey analysis is the development of a realistic everyday work of endoscopy staff. This forms the basis for exploring potential optimizations in terms of simplifying work and improving cost-effectiveness.

The second objective of this descriptive study is to analyze the experiences and concerns of endoscopy nurses regarding the use of single-use and reusable endoscopes.

### Study Design and Participants

Between June and November 2023, a cross-sectional, international, online survey was conducted. The survey was distributed to nurses working in endoscopy departments, primarily in gastroenterology, in 33 countries, and was available in six languages: English, German, Spanish, Italian, French, and Japanese. In May 2023, the survey was approved by the ESGENA governing board and released for distribution via the ESGENA newsletter and emails to national endoscopy nurses’ societies and ESGENA individual members. Two reminders were sent out.

The survey consisted of 23 multiple-choice questions which reflect daily practice based on clinical experience and international guidelines (see supplemental material). In order to proceed to the next question and complete the survey, all questions required an answer. For some questions, more than one response could be selected. The full survey is provided as a supplement.

The survey was designed to determine the clinical workload based on the various work locations (e.g., ICU, OR), patient transportation, moving the equipment, and nonclinical workload (cleaning and reprocessing flexible endoscopes). In the second part of the survey, potential stressors and strain factors due to these tasks were identified.

### Ethical Considerations

Completion of the survey was voluntary, anonymous, and unpaid. Completion and submission of the survey implied the consent of the participant. The survey did not include any patient data.

### Data

Responses were collected using Microsoft Forms. All data were anonymous, with only the country of residence, years of experience, primary work department, and number of beds in the medical center (<100, 100–500, >500) as identifiable characteristics. No unique personal identifiable data were collected.

### Data Analyses

Statistical analysis was performed using RStudio version 4.4.1 (2024-06-14). Data were analyzed using descriptive statistics and additional statistical tests for association between responses. Pearson’s Chi-squared test was used to assess the association between the number of beds at the medical center or the number of annual procedures and the frequency of transport of the tower and of patients. Chi-squared tests were used to assess all perceived stress factors. Pearson’s Chi-squared test with Yate’s continuity correction was used to assess the association between concerns about reusable compared with single-use endoscopes.

## Results

### Participants

A total of 570 responses were received from endoscopy nurses in over 30 countries. The majority of respondents ( *n* = 456) worked in departments focused on gastrointestinal (GI) endoscopy, either alone or in departments where nurses perform both GI endoscopy and bronchoscopy ( Table 1 ). The number working in other departments was low, and it was deemed that these groups were too small to contribute meaningful insights to the analysis. Therefore, the decision was made to focus only on nurses working in GI departments, and statistical analysis was carried out using a total study population of those 456 respondents.

**Table 1:** Medical center information.

Endoscopy procedures performed per year	Number	Percentage
1–500	8	2%
501–1000	17	4%
1001–3000	85	19%
3001–5000	124	27%
5001–7000	93	20%
7001–10,000	65	14%
10,001–15,000	37	8%
>15,000	27	6%
**Total**	**456**	**100%**
**Beds in medical center**		
<100	57	13%
100–500	243	53%
>500	156	34%
**Total**	**456**	**100%**

The majority of respondents ( *n* = 265; 58%) were from Germany, followed by Spain ( *n* = 21; 5%), Italy ( *n* = 18; 4%), and France ( *n* = 16; 4%). The survey was completed by between 8 and 15 participants from each of the following countries: Portugal, Greece, Sweden, Australia, Ireland, and Estonia, accounting for 2–3% of responses from each country.

Most respondents ( *n* = 294; 64%) had more than ten years’ experience in endoscopy, and most specified that their primary work department was GI endoscopy alone ( *n* = 364; 80%).

Most respondents ( *n* = 243; 53%) worked at centers with 100–500 beds, and just under half ( *n* = 217; 48%) reported that their endoscopy unit performed 3001–7000 procedures a year.

### Clinical Tasks

#### Movement of the Endoscopy Tower

The majority of respondents reported that they or their department had to move the endoscopy tower to another department, most frequently moved to the ICU ( *n* = 284; 62%), followed by the OR ( *n* = 98; 21%).

The vast majority of respondents ( *n* = 396; 87%) reported that the endoscopy tower was mostly moved by endoscopy nurses. The frequency with which this was required varied from daily ( *n* = 104; 23%), to weekly ( *n* = 194; 43%), to monthly ( *n* = 92; 20%), to rarely ( *n* = 23; 5%) or never ( *n* = 43; 9%). Generally, this took less than 10 min ( *n* = 242; 53%), with a further *n* = 158 respondents (35%) reporting that it took 10–20 minutes, and 13 respondents (3%) reporting that it took longer.

A statistically significant association was found between the number of beds at the medical center and the need to transport the endoscopy tower. Larger medical centers moved the tower more frequently ( *p* < 0.001). Centers performing a greater number of annual procedures also moved the tower more frequently ( *p* < 0.001).

#### Transport of Patients

Patients were transported from other departments to the endoscopy unit on a daily basis ( *n* = 94; 21%), weekly ( *n* = 232; 51%), and monthly ( *n* = 104; 23%). In medical centers with more than 500 beds, patients are less often transported by endoscopy nurses than in medical centers with fewer beds.

#### Delays in Procedures Due to Lack of Available Endoscopes

**Fig. 1:**
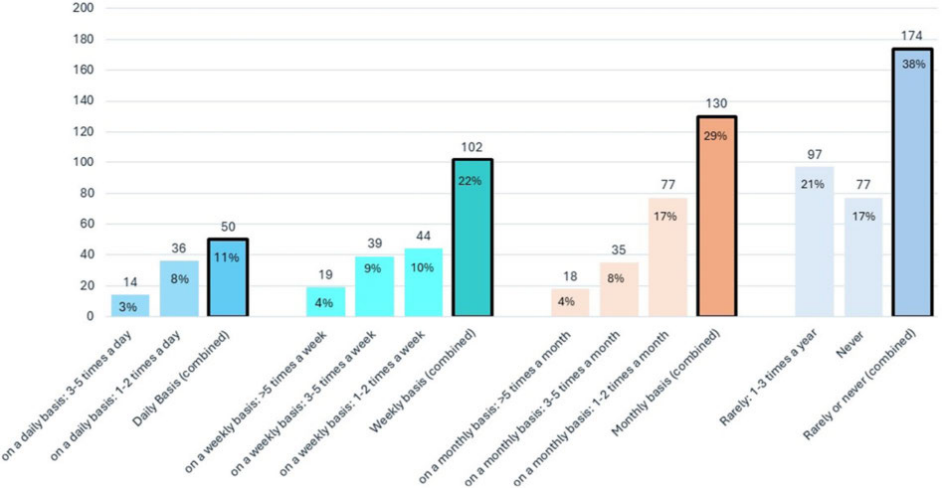
Delay in patient procedures due to lack of ready-to-use endoscopes.

Over half of the respondents reported delays in patient procedures due to a lack of available reusable endoscopes; 50 respondents (11%) reported this on a daily basis, 102 respondents (22%) on a weekly basis, and 130 respondents (29%) on a monthly basis ( Fig. 1 ). Only 77 respondents (17%) reported never experiencing such delays.

### Nonclinical Tasks

#### Endoscope Reprocessing and the Role of Nurses

**Fig. 2:**
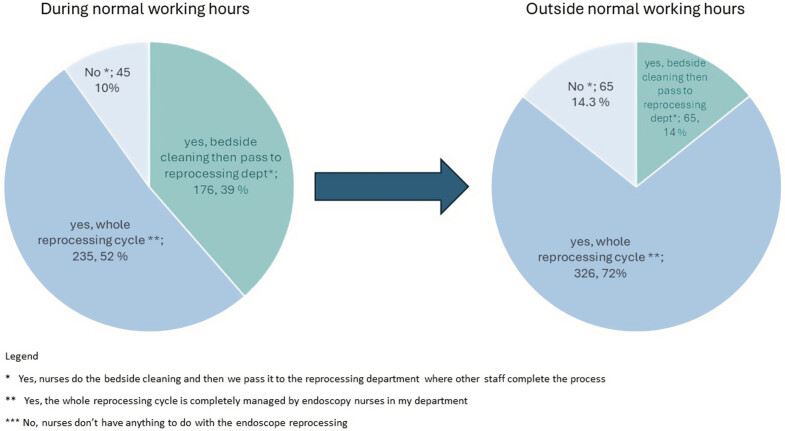
Active role of nurses in the endoscope reprocessing.

Most respondents noted that nurses in the endoscopy department played an active role in endoscope reprocessing. Just over half of the respondents ( *n* = 235; 52%) stated that endoscopy nurses were involved in endoscope reprocessing during regular working hours on weekdays. During on-call services, including nights, weekends, and national bank holidays, this increased to *n* = 326 (72%) ( Fig. 2 ).

#### Stress with Reprocessing

**Fig. 3:**
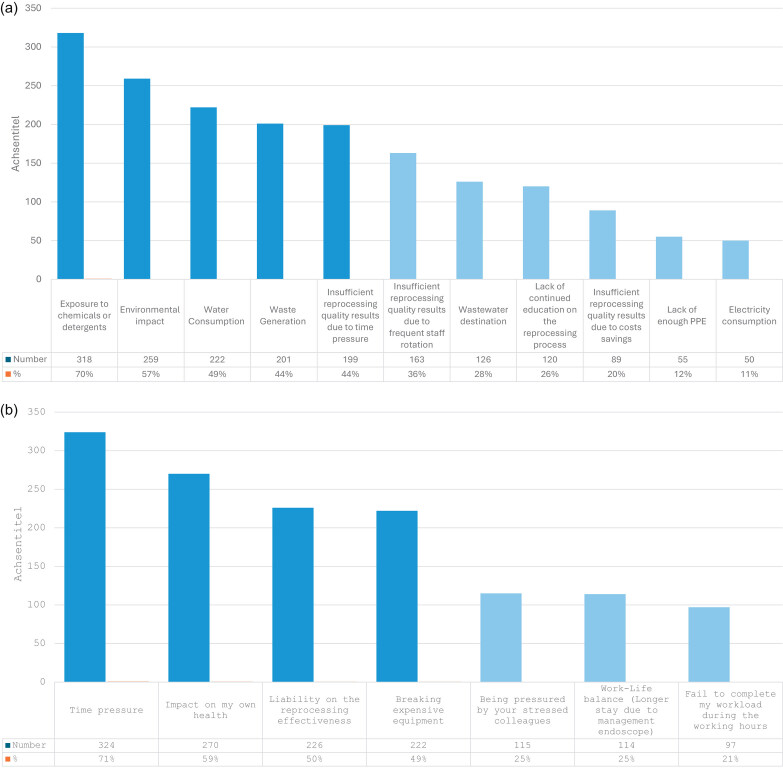
( **a** ) Concerns with the reprocessing process. ( **b** ) Stressful scenarios/factors related to the reprocessing process.

A high proportion of nurses reported concerns about the reprocessing process. The most frequently reported concerns were exposure to chemicals or detergents ( *n* = 318; 70%), environmental impact ( *n* = 259; 57%), water consumption ( *n* = 222; 49%), waste generation ( *n* = 201; 44%), and insufficient reprocessing quality due to time pressure ( *n* = 199; 44%) ( Fig. 3a ). The top four stressful scenarios/factors related to the reprocessing process were time pressure ( *n* = 324; 71%), impact on the respondent’s own health ( *n* = 270; 59%), liability for reprocessing effectiveness ( *n* = 226; 50%), and damage to expensive equipment ( *n* = 222; 49%) ( Fig. 3b ).

Further statistical analysis showed that nurses who did not reprocess endoscopes during their shift were significantly less stressed about their health than nurses who did reprocess endoscopes during their shift ( *p* = 0.01824). Those who performed all parts of the reprocessing were more stressed about their health than those who only performed bedside cleaning ( *p* > 0.05).

#### Experience with Single-use Endoscopes

Experience with single-use endoscopes was limited, with just under half ( *n* = 219; 48%) of the nurses reporting that they had no experience with any single-use endoscopes, although almost one in three ( *n* = 141; 31%) reported experience with single-use bronchoscopes. In all, 18% ( *n* = 83) used single-use cholangioscopes. About 9% ( *n* = 43) had experience with single-use duodenoscopes and 9% ( *n* = 19) with single-use gastroscopes.

Most respondents ( *n* = 284; 62%) reported that they never assist in endoscopy procedures where single-use endoscopes are used, with a further 18% ( *n* = 84) reporting that they do so less than once a month. About 12% ( *n* = 56) assisted in procedures with single-use endoscopes monthly, 4% ( *n* = 18) weekly, and 3% ( *n* = 14) on a daily basis.

**Fig. 4:**
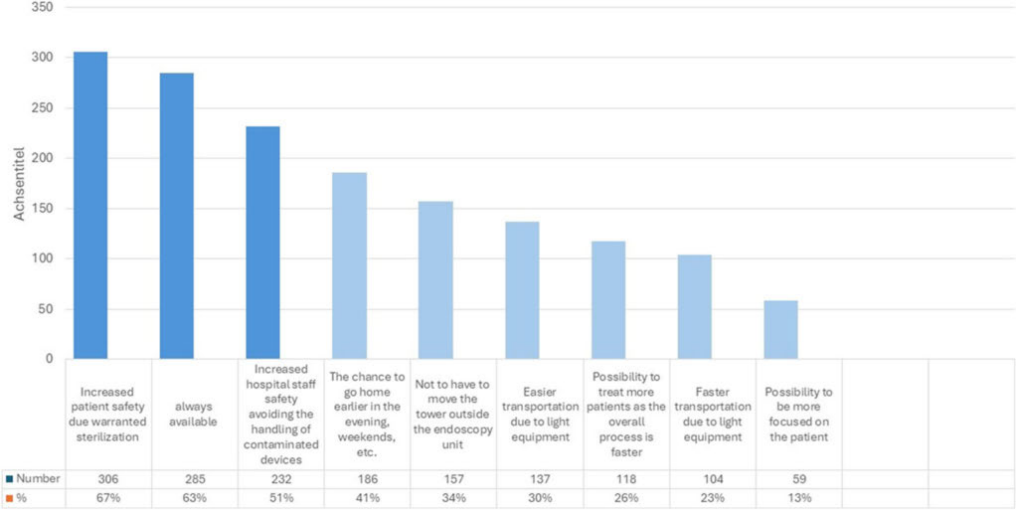
Reported benefits provided by single-use endoscopes on a regular basis.

Despite these relatively low levels of experience, most respondents reported benefits that they thought single-use endoscopes could provide, including most commonly increased patient safety due to warrantied sterilization ( *n* = 306; 67%), endoscopes being always available ( *n* = 285; 63%) and increased hospital staff safety avoiding the handling of contaminated devices ( *n* = 232; 51%) ( Fig. 4 ).

#### Concerns with Single-use vs. Reusable Endoscopes

**Fig. 5:**
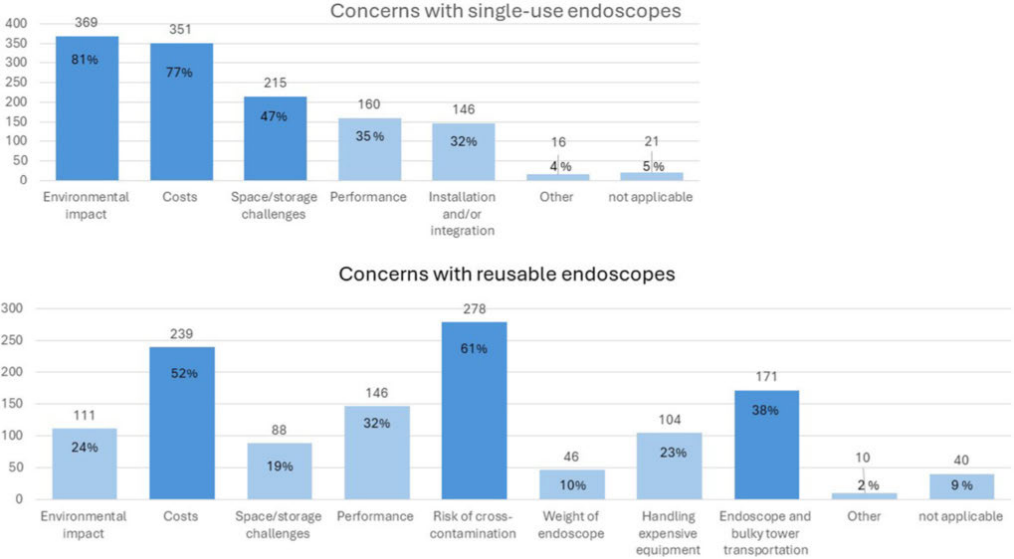
Concerns with single-use vs. reusable endoscopes.

The nurses were asked about their concerns regarding single-use and reusable endoscopes. Cost was a frequent concern for both types, with 77% and 52% of respondents selecting this option for single-use and reusable endoscopes, respectively. For single-use endoscopes, the three-most frequently selected concerns were environmental impact ( *n* = 369; 81%), cost ( *n* = 351; 77%), and space/storage challenges ( *n* = 215; 47%). For reusable endoscopes, the three-most frequently cited concerns were the risk of cross-contamination ( *n* = 278; 61%), cost ( *n* = 239; 52%), and transportation of the endoscope and bulky tower ( *n* = 171; 38%) ( Fig. 5 ).

Of the four concerns asked about for both types of endoscopes (environmental impact, cost, space/storage challenges and performance), the differences between single-use and reusable endoscopes were not statistically significant.

## Discussion

This survey of endoscopy nurses aimed to further understand workflows and practices in endoscopy units in order to identify potential areas to improve efficiency. It was shown that nonclinical tasks were being carried out by nurses at a notable frequency.

### Transport of Equipment

Emergency endoscopies and interventions on critically ill patients are frequently performed in ICUs, emergency departments, or ORs, requiring the transport of endoscopy-specific equipment. In 43% of the facilities, these external procedures occurred weekly, and in 23% of the departments, even daily. No data are available on the weight of endoscopy equipment transported daily by endoscopy personnel for external examinations. There is likely to be a wide variation in weights, due to the differing endoscopy tower specifications between hospitals. Nevertheless, the weight and size of the single-use endoscope processor is significantly lower and smaller than the conventional endoscopy towers. Larger medical centers may also see greater cost savings, since in this survey there were statistically significant associations between the number of beds at the medical center as well as the number of annual procedures and the need to transport the endoscopy tower, with larger centers and those with a higher number of annual procedures moving the tower more frequently.

### Delays

The majority of participating nurses (60%) reported delays to patient procedures due to a shortage of reusable endoscopes. This shortage of ready-to-use endoscopes can be caused by lack of reprocessing staff, lack of endoscope washer disinfectors, and consequently delayed reprocessing. It can also be caused by insufficient number of endoscopes in relation to the number of scheduled patients. Such delays can generate costs, reduce the quality of care, and affect treatment outcomes. 2 For example, an analysis of over 7000 screening colonoscopes found a significant association between delays of one to two hours and a reduced adenoma detection rate. 3

### Reprocessing

Traditionally, endoscope reprocessing is performed in centralized reprocessing units, which are located within the endoscopy units. The majority of respondents to this survey reported being involved in endoscope reprocessing, either by carrying out bedside cleaning or by being responsible for the entire reprocessing cycle. Endoscope reprocessing is time-consuming and labor-intensive, incurring both direct and hidden costs. Reprocessing is a complicated process involving complex cleaning procedures, staff training, waste disposal, and machine repairs. 4 For example, reprocessing a reusable duodenoscope can involve more than 100 individual steps. 5 When endoscopy nurses spend their time on endoscope reprocessing, this equates to paid hours spent performing nonclinical tasks. Alternatively, staff from CSSD departments or specially trained staff can release nurses from endoscope reprocessing. The survey confirms this practice. According to the survey results, nurses were more often responsible for the entire reprocessing cycle outside of normal working hours, which can increase costs in the form of overtime and workplace stress. Not all hospitals have specially trained staff for endoscope reprocessing available out of hours, meaning nurses may need to carry out the reprocessing themselves or additional staff must be called in – potentially increasing stress, costs, and requiring greater organizational efforts.

### Stress and Health Risks

Nurses also expressed various concerns about the reprocessing process itself, including exposure to chemicals and the poor quality of reprocessing. The health risks associated with reprocessing chemicals lead to increased stress among nurses who perform reprocessing more frequently. Reduced job satisfaction and increased stress can negatively impact staff retention. 6 However, this value is difficult to quantify and can vary depending on the individual and location. Nevertheless, such stressors may contribute to the staffing problems reported in many healthcare facilities in recent years. 7

### Endoscopy-related Infections

A potential risk of infection is inherent with all endoscopies, 4 8 although evaluating the precise level of risk is challenging due the difference and nature of endogenous and exogenous infections. 8 There is also a risk of cross-contamination between patients. If an infection occurs, further direct costs can arise due to the need for additional treatment. Single-use endoscopes are sterile and add an extra measure of safety for the most vulnerable patients, preventing the spread of pathogens from patients with high-risk infections.

### Single Use

One way to reduce the time spent on nonclinical tasks is to integrate single-use endoscopes into selected clinical and workflow-related procedures. Single-use endoscopes can simplify workflows, improve infection prevention, and reduce costs for reprocessing, maintenance, and logistics and are already widely used in ICU and in anesthesiology. Single-use endoscopes can also provide greater immediate availability and reduce delays in procedures due to unplanned downtime, which may be a significant benefit given that the majority of respondents to this survey reported seeing such delays. A US study found that respondents estimated using single-use endoscopes freed up at least four hours per week for other tasks, while a separate study found single-use duodenoscopes had significantly faster changeover times than reusable ones. 9 10 Staff also reported high satisfaction levels with single-use duodenoscopes in terms of factors such as technical performance, cleaning and reprocessing, and environmental impact. 10

### Green Endoscopy

The majority of respondents in this study highlighted the environmental impact of single-use endoscopes as a concern. A quarter of respondents cited environmental concerns associated with reusable endoscopes. In practice, accurately comparing the overall environmental impact of single-use and reusable endoscopes is complex as it is challenging to quantify the environmental burden of production, potential for recycling, the reprocessing procedure, and need for repairs and replacement. 8 11 12 These concerns may also be partly due to the great amount of waste generated in gastrointestinal procedures due to the broad use of single-use accessories. Programs to improve recycling of single-use endoscopes, increase the use of recycled materials with a lower carbon footprint, and increase the recycling of components have been described elsewhere. 5 13 The European Society of Gastrointestinal Endoscopy (ESGE) and the European Society of Gastroenterology and Endoscopy Nurses and Associates (ESGENA) recommend embedding reduce, reuse, and recycle programs into GI endoscopy units, support the promotion and funding of research into “green and sustainable” procedures, and recommend conducting high-quality research to quantify and minimize the environmental impact of GI endoscopy. 8

### Cost-effectiveness

The majority of respondents stated that costs are an issue for both single-use and reusable endoscopes. The following calculation can be used to estimate personnel costs in Germany: the average monthly salary of an endoscopy nurse in Germany is €4394 for a 40-hour week. 14 This equates to an estimated average hourly wage of €25.35. Slightly more than half (51%) of nurses said they were involved in the entire reprocessing process. Outside of working hours, this figure increased to 72%. Reprocessing time is estimated at 59 minutes, 23 of which are hands-on (not including the time the endoscope spends in the cleaning machine). 12 15 Assuming an average of 806 GI endoscopies per year (based on the average number per hospital in Germany in 2023 16 ) and one nurse per procedure, the cost of nursing effort for reprocessing in an average endoscopy unit per year is €7832.31 *(*Calculated as (23/60 × €25.35 × 806; hours, cost per hour, procedures per year)). Although these costs are only approximate, they provide insight into the impact of reprocessing on overall costs and the potential utilization of qualified nursing staff for non-clinical tasks. Reprocessing requires a special training, which is mandatory in some European countries like Germany. If nurses mainly reprocess their endoscopes during emergency procedures in on-call services, these training costs also have to calculated while using reusable endoscopes. Other reprocessing costs include materials, energy, and water, and these would need to be considered in a comprehensive cost–benefit analysis. Improving the efficiency of other processes, such as patient transport, could also lead to significant long-term cost savings. 17 Optimizing workflows could free up clinical hours for additional procedures without placing extra strain on nursing staff.

In a joint statement, ESGE-ESGENA recommended that the use of single-use GI endoscopes be considered on an individual basis for selected patients. 8 When assessing the benefits and costs of single-use versus reusable endoscopes, and determining when to use each type, other factors must be considered, including the indirect costs associated with endoscopy. In practice, a hybrid strategy using both types of endoscopes may be the most efficient for endoscopy departments in terms of benefits and risks/costs. Settings where urgent access to endoscopes is required due to emergencies outside regular working hours or an endoscope is needed outside the endoscopy unit may benefit from the immediate availability of single-use endoscopes.

### Limitations of This Study

This study has some limitations, as it was an online survey with predefined questions and response options and was not formally validated. Endoscopy nurses from 33 countries participated in the survey. Practices may vary between countries and medical centers, and the results may not be representative of other locations. Participation in the survey was voluntary, so response bias may have occurred. However, the sample size was large (over 400 individuals) and the survey was distributed through the ESGENA professional network.

## Conclusion

Responses to this survey suggest that GI endoscopy nurses dedicate a substantial portion of their time to nonclinical activities, such as transporting endoscopy towers and reprocessing. The reprocessing process can be time-consuming and stressful for staff. A review of working practices in endoscopy units should be conducted to identify potential cost and efficiency savings, such as the targeted use of single-use endoscopes where appropriate.

## Notes

Note This article was changed according to the Correction on July 13, 2026.
**Correction**
In the above-mentioned article, corrections have been made to Figures 2 and 5 and to the paragraph entitled “Transport of Patients”. This was corrected in the online version on July 13, 2026.

## Supplementary Material

Ergänzendes Material Supplementary Material
