# Correction: Hidden Costs of Workflow Challenges in Endoscopy Units: Insights from a Multinational Survey

**DOI:** 10.1055/a-2908-9884

**Published:** 2026-07-15

**Authors:** Ulrike Beilenhoff, Christoph Schlag, Dörte Wichmann

**Affiliations:** 1 Scientific SecretaryEuropean Society of Gastroenterology and Endoscopy Nurses and Associates (ESGENA)UlmBWGermany; 2 Department of Gastroenterology and Hepatology 27243 UniversitätsSpital ZürichZürichZHSwitzerland; 3 Working group of expertimental endoscopy training and developement, University of Tübingen 9188 University of TübingenTübingenBWGermany



In the above-mentioned article, corrections have been made to Figures 2 and 5 and to the paragraph entitled “Transport of Patients”. This was corrected in the online version on July 13, 2026.

