# Supplementary material for: Hidden Costs of Workflow Challenges in Endoscopy Units: Insights from a Multinational Survey
Source: Endosc Int Open. 2026 Jun 10;14:a28797529. doi: 10.1055/a-2879-7529 (PMC13289772; doi:10.1055/a-2879-7529)
Supplement: Supplementary file 1 — Ergänzendes Material [file 10-1055-a-2879-7529_28819242.pdf]

Supplementary material

Ulrike Beilenhoff, Christoph Schlag , Dörte Wichmann

Hidden costs of workflow challenges in endoscopy units: insights from a multinational survey

Survey questions

1. **Country of residence**

2. **How many years experience do you have in endoscopy?**
   - Less than 2
   - Between 2 and 5
   - Between 6 and 8
   - Between 7 and 10
   - More than 10

3. **Please specify your primary work department**
   - GI Endoscopy Department
   - Bronchoscopy Department
   - Urology Department
   - Ear Nose Throat (ENT) Department
   - Women's Health
   - Other

4. **On average, how frequently do you/your endoscopy department move the endoscopy tower to another department (e.g.: ICU, emergency department, OR)?**
   - On a daily basis: Between 3 to 5 times a day
   - On a daily basis: Between 1 and 2 times a day
   - On a weekly basis: More than 5 times a weeks
   - On a weekly basis: Between 3 to 5 times a week
   - On a weekly basis: Between 1 and 2 times a week
   - On a monthly basis: More than 5 times a month
   - On a monthly basis: Between 3 to 5 times a month
   - On a monthly basis: Between 1 and 2 times a month
   - Rarely: Between 1-3 times a year
   - Never because the endoscopy tower is already installed in place
   - Never because endoscopies are never performed outside the endoscopy unit

Endosc Int Open | DOI 10.1055/a-2879-7529 | © The Author(s).

5. **Please specify, on average, how long it takes to transport the endoscopy tower from the Endoscopy Unit to another department where the endoscope is needed:**
   - Less than 10 min
   - Between 10 to 20 min
   - Between 21 to 40 min
   - Between 41 to 60 min
   - More than 60 min
   - Not applicable

6. **Please re-order the departments below based on how frequently you normally move the endoscopy tower to that department**
   - Intensive Care Unit (ICU)
   - Operating Room (OR)
   - Emergency Department
   - Others
   - Not Applicable

7. **Is the endoscopy tower mostly moved by the endoscopy nurses?**
   - Yes
   - No

8. **On average, how frequently are patients transported from other departments (ICU, Emergency Department, OR) to the Endoscopy Unit?**
   - On a daily basis: More than 5 times a day
   - On daily basis: Between 3 to 5 times a day
   - On a daily basis: Between 1 and 2 times a day
   - On a weekly basis: More than 5 times a weeks
   - On a weekly basis: Between 3 to 5 times a week
   - On a weekly basis: Between 1 and 2 times a week
   - On a monthly basis: More than 5 times a month
   - On a monthly basis: Between 3 to 5 times a month
   - On a monthly basis: Between 1 and 2 times a month
   - Never

9. **During normal working hours on the weekdays, do the nurses in your endoscopy department have an active role in the endoscope reprocessing process?**
   - Yes, we do the bedside cleaning (first working channel flushing and external wiping just right after the procedure) and then we pass it to the reprocessing department where other staff complete the process
   - Yes, the whole reprocessing cycle is completely managed by endoscopy nurses in my department
   - No, we don't have anything to do with the endoscope reprocessing

Endosc Int Open | DOI 10.1055/a-2879-7529 | © The Author(s).

10. **Outside normal working hours (e.g. nights, weekends, national holidays), do the nurses in your endoscopy department have an active role in the endoscope reprocessing process?**
    - Yes, we do the bedside cleaning (first working channel flushing and external wiping just right after the procedure) and then we pass it to the reprocessing department where other staff complete the process
    - Yes, the whole reprocessing cycle is completely managed by endoscopy nurses in my department
    - No, we don't have anything to do with the endoscope reprocessing

11. **On average, how often do you see delay in patient procedures because there are no reusable endoscopes available for that specific case?**
    - On daily basis: Between 3 to 5 times a day
    - On a daily basis: Between 1 and 2 times a day
    - On a weekly basis: More than 5 times a week
    - On a weekly basis: Between 3 to 5 times a week
    - On a weekly basis: Between 1 and 2 times a week
    - On a monthly basis: More than 5 times a month
    - On a monthly basis: Between 3 to 5 times a month
    - On a monthly basis: Between 1 and 2 times a month
    - Rarely: Between 1 and 3 times a year
    - Never

12. **Could you please indicate what types of single-use endoscopes you have been exposed to the most? Please mark all that apply.**
    - Single-use Gastroscopes
    - Single-use Duodenoscopes
    - Single-use Colonoscopes
    - Single-use Cholangioscopes/Choledochoscopes
    - Single-use Bronchoscopes
    - Single-use Rhino-laryngoscopes
    - Single-use Cystoscopes
    - Single-use Ureteroscopes
    - Single-use Nephroscopes
    - I don't have experience with any single-use endoscopes

13. **How often do you assist endoscopy procedures where single-use endoscopes are used?**
    - Several times a day
    - Once a day
    - More than once a week
    - Once a week
    - Several times a month
    - Once a month
    - Less than once a month
    - Never

Endosc Int Open | DOI 10.1055/a-2879-7529 | © The Author(s).

14. Which benefits do you think single-use endoscopes provide on a regular basis? Please mark all that apply.

Always available

- Possibility to be more focused on the patient
- Possibility to treat more patients as the overall process is faster
- The chance to go home earlier in the evening, weekends, etc.
- Increased patient safety due warranted sterilization
- Increased hospital staff safety avoiding the handling of contaminated devices
- Easier transportation due to light equipment
- Faster transportation due to light equipment
- Not to have to move the tower outside the endoscopy unit (In case a fixed processor is placed in the needed department, outside the endoscopy unit)

15. Please re-order the departments below based on where you see most advantages for single-use endoscopes

- Intensive Care Unit (ICU)
- Operating Room (OR)
- Emergency Department
- Others
- Not Applicable

16. What concerns do you have with single-use endoscopes? Please mark all that apply.

- Environmental impact
- Cost
- Space/storage challenges
- Performance
- Installation and/or integration with available endoscopy equipment
- Other
- Not Applicable

17. If your answer was "other" could you specify which one?

18. Which concerns do you have with reusable endoscopes? Please mark all that apply.

- Environmental impact
- Cost (Capital investments, maintenance and repair costs, reprocessing costs, etc.)
- Space/storage challenges
- Performance due to deterioration
- Risk of cross-contamination
- Weight of the endoscope
- Handling expensive equipment
- Endoscope and bulky tower transportation
- Other
- Not Applicable

Endosc Int Open | DOI 10.1055/a-2879-7529 | © The Author(s).

19. **If your answer was "other" could you specify which one?**

20. **What concerns do you or your colleagues have with the reprocessing process? Mark all that apply.**
    - Exposure to chemicals or detergents
    - Lack of enough PPE (Personal Protective Equipment)
    - Environmental impact
    - Waste generation
    - Water consumption
    - Wastewater destination
    - Electricity consumption
    - Insufficient reprocessing quality results due to frequent staff rotation
    - Insufficient reprocessing quality results due to time pressure
    - Insufficient reprocessing quality results due to costs savings
    - Lack of continued education on the reprocessing process

21. **Please select the TOP 3 stressful scenarios/factors related to the reprocessing process?**
    - Impact on my own health
    - Time pressure
    - Being pressured by your stressed colleagues
    - Fail to complete my workload during the working hours
    - Work-Life balance (Longer stay due to management endoscope)
    - Liability on the reprocessing effectiveness
    - Breaking expensive equipment

22. **How many beds does your medical center have?**
- Fewer than 100 beds
- Between 100 to 500 beds
- More than 500 beds

23. **Approximately how many endoscopy procedures are performed in your endoscopy unit per year? (Including all type of endoscopies)**
- 1 - 500
- 501 – 1.000
- 1.001 – 3.000
- 3.001 – 5.000
- 5.001 – 7.000
- 7.001 – 10.000
- 10.001 – 15.000
- More than 15.000

Endosc Int Open | DOI 10.1055/a-2879-7529 | © The Author(s).
